# Alternating and Concurrent True Hyperkalemia and Pseudohyperkalemia in Adult Sickle Cell Disease

**DOI:** 10.5041/RMMJ.10439

**Published:** 2021-04-29

**Authors:** Macaulay Amechi Onuigbo, Heng Tan, Sarah E. Sherman

**Affiliations:** The Robert Larner, M.D. College of Medicine, University of Vermont, Burlington, Vermont, USA

**Keywords:** Hyperkalemia, plasma, pseudohyperkalemia, serum, sickle cell disease, sickle cell nephropathy, thrombocytosis

## Abstract

Sickle cell disease (SCD) predisposes the patient to recurrent episodes of acute painful hemolytic crisis. Sickle cell nephropathy (SCN) is not uncommon in adult patients, and renal manifestations of SCN include renal ischemia, microinfarcts, renal papillary necrosis, and renal tubular abnormalities with variable clinical presentations. Intravascular hemolysis and reduced glomerular filtration rate with renal tubular dysfunction predispose to true hyperkalemia. Hemolytic crisis can be complicated by sepsis, leading to significant degrees of thrombocytosis, and thrombocytosis is a well-defined cause of pseudohyperkalemia. We describe a 40-year-old African American male patient with sickle cell anemia who exhibited alternating episodes of true hyperkalemia and pseudohyperkalemia, during consecutive hospital admissions. Clearly, true hyperkalemia is a potentially lethal condition. At the same time, the institution of inappropriate and intensive treatment of pseudohyperkalemia leading to severe hypokalemia is also potentially lethal. The need for this caution is most imperative with the recent introduction of the safer and more potent potassium binders, patiromer and sodium zirconium cyclosilicate.

## BACKGROUND

Sickle cell disease (SCD) predisposes the patient to recurrent episodes of acute painful hemolytic crisis. Sickle cell nephropathy (SCN) is not uncommon in adult patients. The presence of sickled erythrocytes in the renal medullary vessels is the hallmark of the disease, and renal manifestations of SCN include renal ischemia, microinfarcts, renal papillary necrosis, and renal tubular abnormalities with variable clinical presentations.^1^ Furthermore, acute hemolytic crisis can be complicated by sepsis.^2^ Moreover, sepsis is a potent cause of thrombocytosis. Hemolysis, specifically intravascular hemolysis, can produce true hyperkalemia.^1^ Additionally, reduced glomerular filtration rate from SCN predisposes to true hyperkalemia.^3^,^4^ Pseudohyperkalemia was first reported by Hartmann and Mellinkoff in 1955 as a marked elevation of serum potassium levels in the absence of clinical evidence of electrolyte imbalance.^5^ In pseudohyperkalemia, simultaneously estimated serum potassium exceeds plasma potassium by >0.4 mmol/L.^6^ This is often associated with moderate to severe thrombocytosis or leukocytosis.^7^,^8^ Clearly, true hyperkalemia is a potentially lethal condition. At the same time, the institution of inappropriate intensive treatment of pseudohyperkalemia leading to severe hypokalemia is also potentially lethal. We describe a 40-year-old African American male patient with sickle cell anemia who exhibited alternating episodes of true hyperkalemia and pseudohyperkalemia, during consecutive hospital admissions.

## CASE REPORT

**Figure 1:**
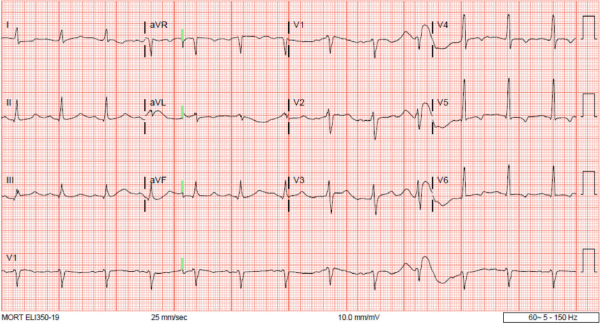
Abnormal EKG Tracings Abnormal EKG tracings in the ED during the first hospitalization with acute kidney injury, metabolic acidosis, and true hyperkalemia of 7.4 mmol/L.

A 40-year-old African American male patient, known to have homozygous sickle cell disease (SS genotype) and associated nephropathy, was admitted to our medical service in the summer of 2020 with a painful hemolytic crisis. He had presented with several weeks of progressive generalized weakness, generalized myalgias, and worsening dyspnea in the previous week. He had failed to take his prophylactic hydroxyurea for some time prior to presentation. Initial evaluation in the emergency department (ED) revealed lethargy and sedated level of consciousness with hypoxia and increased work of breathing. Following initial resuscitative measures in the ED, he improved. Pertinent admission laboratory data included sickle hemoglobin (HbS) 94.5%, hemoglobin A2 3.2%, mean corpuscular volume 103 fL, reticulocyte count 19.4% (baseline reticulocyte count of ~2.8%), white blood cell count 34.85×10^9^/L with 85% neutrophils, platelet count 294×10^9^/L, hemoglobin 4.6 g/dL, and hematocrit 13.1%. Peripheral smear revealed sickled red blood cells (RBCs). Chemistry revealed sodium 136 mmol/L, potassium 7.4 (3.5–5.0) mmol/L, glucose <20 mg/dL that quickly improved after 10% dextrose infusion, creatinine 7.20 mg/dL (baseline 1.5 mg/dL), bicarbonate <5 mmol/L, chloride 102 mmol/L, calcium 7.4 mg/dL, total bilirubin >40 mg/dL, phosphorus 11.6 mg/dL, conjugated bilirubin 38.7 mg/dL, unconjugated bilirubin 2.9 mg/dL, aspartate aminotransferase 374 IU/L, alanine aminotransferase 94 IU/L, alkaline phosphatase 418 IU/L, total protein 10.7 g/dL, albumin 3.6 g/dL, ferritin >6,000 ng/mL, creatine kinase 70 U/L, troponin I 0.042 (<0.034) ng/mL, ammonia 54 (<34) μmol/L, lactate dehydrogenase 3288 (313–618) U/L, N-terminal pro b-type natriuretic peptide 8940 (<125) pg/mL, partial thromboplastin time 31 s, prothrombin time 20.9 s, international normalized ratio 1.8, and lactic acid 11.6 mmol/L. The calculated MELD score was 40. Salicylate level was 1.7 mg/dL, and acetaminophen level was 14 μg/mL. Initial venous blood gas examination revealed pH 7.03, pCO2 38 mmHg, pO2 34 mmHg, TCO2 11 mmol/L, base deficit 19, and oxygen saturation was only 42%. Electrocardiogram (EKG) in the ED was abnormal and demonstrated sinus rhythm with prolonged QTc duration of 522 (<440) ms, together with inverted T waves in the lateral chest leads, V4–V6, consistent with true hyperkalemia (Figure 1). He had promptly received emergency therapies for hyperkalemia including intravenous (IV) calcium gluconate infusion, IV sodium bicarbonate infusion, IV 10% dextrose infusion with insulin, and IV furosemide. In addition, he was started empirically on IV antibiotics, vancomycin, and meropenem.

He was transfused with two units of packed RBCs and was promptly transferred to the Medical ICU. He quickly underwent emergency RBC exchange with therapeutic apheresis via a right femoral vein cannula, utilizing 1,999 mL of packed RBCs with a net fluid balance of 270 mL. Sickle hemoglobin (HbS) quickly dropped to 26.3% post-apheresis exchange. Initial nephrology consultation on admission had recommended correction of metabolic acidosis and treatment of hyperkalemia with more isotonic sodium bicarbonate infusions, insulin/glucose infusions, and the initiation of oral sodium zirconium cyclosilicate (SZC). He received one hemodialysis treatment on day 3 following evidence of encephalopathy which was subsequently blamed on drug toxicity from IV ketamine that was used for pain management.

**Figure 2:**
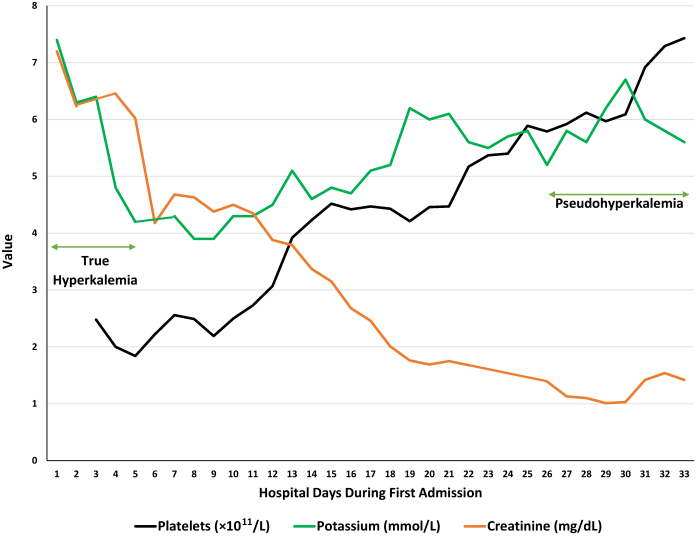
Platelet Count, Blood Potassium, and Serum Creatinine Simultaneous trajectories of platelet count, blood potassium, and serum creatinine during the first hospitalization.

**Figure 3:**
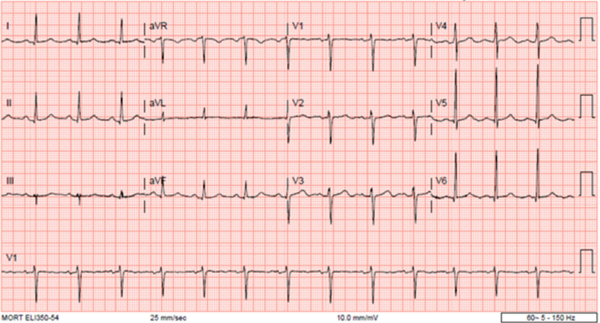
Normal-Appearing EKG Tracings Normal-appearing EKG tracings during the 4th week of the first hospitalization despite a measured serum potassium of 6.7 mmol/L.

Despite observing significant improvement of the acute kidney injury on chronic kidney disease in the first two weeks of this admission with much improved serum creatinine 2–3 weeks into the admission, as well as normal urine output, and stabilization of serum bicarbonate level to 22–27 mmol/L, the patient had exhibited persistent hyperkalemia (5.2–6.7 mmol/L) even with continued administration of SZC at 10 mg 2 times daily. The simultaneous trajectories of serum creatinine, platelet count, and serum potassium concentrations during the admission are shown in Figure 2. The patient had remained otherwise asymptomatic. Chlorthalidone, 25 mg daily, was added to manage the hyperkalemia without much success. Moreover, an EKG obtained in the 4th week of admission when the serum potassium that morning was 6.7 mmol/L was remarkably normal (Figure 3). At this point, as demonstrated in Figure 2, our patient had subsequently developed significantly progressive thrombocytosis, the result of previous treated sepsis. Admission leukocytosis of 34.85×10^9^/L had decreased to 10.67×10^9^/L in the 4th week of this admission. Nevertheless, usual therapies for hyperkalemia were again reinstituted in addition to the continued use of SZC and chlorthalidone.

From the foregoing observations and considerations, especially given the normal EKG shown in Figure 3 compared to the admission EKG in Figure 1 when he had true hyperkalemia with acute kidney injury and metabolic acidosis, for the very first time since this admission the possibility of pseudohyperkalemia was entertained. Consequently, later that same afternoon, following a conference call between the Nephrology and Internal Medicine Services, we repeated the simultaneous serum and plasma potassium measurements (after the emergency therapies for true hyperkalemia had been administered earlier that morning for a measured serum potassium level of 6.7 mmol/L). The measured simultaneous blood potassium levels were 5.8 mmol/L in serum versus 5.2 mmol/L in plasma. The reference range for blood potassium is 3.5–5.0 mmol/L. This confirmed the presence of pseudohyperkalemia secondary to the prevailing progressive thrombocytosis during this later part of the admission (Figure 2).^6^ Pseudohyperkalemia is diagnosed when the serum potassium concentration exceeds that of plasma by at least 0.4 mmol/L.^6^ Subsequently, the new potassium binder SZC was discontinued.

Five weeks after discharge, the patient was re-admitted to the same medical service this time with symptomatic painful sickle cell anemia crisis, fevers, cough with green sputum production, leukocytosis of 31.98×10^9^/L, bilirubin 8.4 mg/dL, and chest radiograph that demonstrated bilateral multifocal patchy infiltrates consistent with pneumonia. Both HIV 1 and 2 and repeated COVID-19 RT-PCR tests were negative. He was started empirically on IV ceftriaxone, IV azithromycin, and IV vancomycin. Blood cultures grew methicillin-resistant *Staphylococcus aureus* within 15 hours in two out of two bottles, and IV antibiotics were de-escalated to 6 weeks of IV vancomycin administration. Admission serum creatinine was only slightly increased at 1.48 mg/dL, potassium was 5.7 mmol/L, but bicarbonate was 23 mmol/L. The patient responded well to IV antibiotics with resolution of the fever, and WBC had quickly dropped to 14.99×10^9^/L after 2 days. Despite this quick overall clinical improvement, persistent hyperkalemia was again recorded throughout this second admission despite a low-potassium diet, IV furosemide, and the addition of oral fludrocortisone, 0.2 mg daily. Admission platelet count of 546 ×10^9^/L had decreased to 314–450×10^9^/L following IV fluids and IV antibiotics. This time round, simultaneous serum and plasma potassium levels were checked multiple times, and serum and plasma potassium concentrations were similar, indicative of true hyperkalemia. Hyperkalemia in the past had been related to pseudohyperkalemia associated with thrombocytosis. Factors contributory to true hyperkalemia were suspected to include sepsis accompanying the sickle cell crisis and intravascular hemolysis, concurrent exposure to heparin (subcutaneous enoxaparin 40 mg daily for deep vein thrombosis prophylaxis), and underlying sickle cell nephropathy with chronic kidney disease. The patient was restarted on SZC 10 g b.i.d. for 48 hours, and continued at 10–15 g daily. Serum potassium was maintained in the 5.3–6.3 mmol/L range throughout the hospital stay. The administration of SZC was to be continued post-discharge for 6 weeks with monitoring of serum potassium levels. To our knowledge, the patient has remained stable since discharge from the hospital.

## DISCUSSION

We believe that this is the first report of adult SCD demonstrating alternating cycles of true hyperkalemia and pseudohyperkalemia at different times, even during one admission. In the first hospitalization, admission true hyperkalemia (7.4 mmol/L) with EKG changes (Figure 1) was clearly secondary to acute kidney injury (serum creatinine of 7.2 mg/dL) and associated anion-gap metabolic acidosis/lactic acidosis with admission venous blood gas pH of 7.03 and serum bicarbonate of <5 mmol/L. Fur-thermore, during the same hospitalization, as he improved and his kidney function had reverted back to normal with resolution of the lactic/metabolic acidosis and the control of sepsis, high potassium levels from true hyperkalemia which had resolved earlier had now started to rise again despite the use of diuretics and the addition of SZC, a new potassium binder (Figure 2). Given the confounding findings of almost normalized kidney function, resolved metabolic acidosis, and a normal-appearing EKG, the possibility of pseudohyperkalemia was then entertained for the first time. The simultaneous serum versus plasma potassium measurements confirmed the presence of pseudohyperkalemia, and hence SZC was discontinued at that point in the first hospitalization. Pseudohyperkalemia was secondary to thrombocytosis which peaked at a level of 743 ×10^9^/L during this hospitalization; this would explain the normal-appearing EKG despite a previously recorded serum potassium of 6.7 mmol/L that morning (Figure 3). The WBC count was not elevated to contribute to the pseudohyperkalemia.

Nevertheless, during the second consecutive admission, the picture regarding elevated potassium levels was more complicated, but again the possibility of pseudohyperkalemia was revisited as the cause of persistent elevated potassium levels. However, this time round, simultaneous testing supported true hyperkalemia, and SZC administration was initiated. The recorded data from this patient seem to suggest that it is indeed possible for both true hyperkalemia and pseudohyperkalemia to coexist simultaneously in a patient at one point in time. Actually, the diagnoses of hyperkalemia and pseudohyperkalemia need not necessarily be mutually exclusive of each other. Thus, during the first admission, although we had confirmed the diagnosis of pseudohyperkalemia secondary to ambient thrombocytosis, the plasma potassium concentration of 5.2 mmol/L, higher than reference range, was still therefore consistent with mild true hyperkalemia.

We must draw attention to the availability of the new potassium binders, patiromer and sodium zirconium cyclosilicate.^9^ We would advocate for caution in the use of these potent potassium binders and to always give consideration to the presence of pseudohyperkalemia under appropriate clinical scenarios.^6^–^8^,^10^ We posit that providers managing adult patients with sickle cell disease must be aware of such a phenomenon to avoid the dangers of over-treatment of episodes of pseudohyperkalemia in such patients.^10^

In conclusion, we suggest that the diagnoses of both true hyperkalemia and pseudohyperkalemia concurrently present in a patient at one point in time must be considered as indeed possible as demonstrated by our patient during the latter part of the first admission. Arguably, these two conditions do not necessarily have to be mutually exclusive of each other. This is the first time that true hyperkalemia and pseudohyperkalemia have been described as concurrently present in one patient, at one point in time. Our understanding of hyperkalemia—true hyperkalemia as well as pseudohyperkalemia—continues to evolve.

## Abbreviations

COVID-19: coronavirus disease 2019
ED: emergency department
EKG: electrocardiogram
HbS: sickle hemoglobin
HIV: human immunodeficiency virus
IV: intravenous
RBCs: red blood cells
SCD: sickle cell disease
SCN: sickle cell nephropathy
SZC: sodium zirconium cyclosilicate.
